# Seroprevalence of *Taenia solium* and *Trichinella spiralis* among Humans and Pigs in Ghana

**DOI:** 10.1155/2021/1031965

**Published:** 2021-10-08

**Authors:** Henry Ofosu Addo, Ayodele O. Majekodunmi, Eric Sampane-Donkor, Lawrence Henry Ofosu-Appiah, David Opare, Godfred Owusu-Okyere, Kingsley Ebenezer Amegah, Langbong Bimi

**Affiliations:** ^1^Department of Animal Biology and Conservation Science, University of Ghana, Ghana; ^2^Livestock and Poultry Research Centre, College of Basic and Applied Sciences, University of Ghana, Ghana; ^3^Department of Medical Microbiology, University of Ghana, Ghana; ^4^National Public Health Reference Laboratory, Korle-Bu, Ghana; ^5^Hohoe Municipal Hospital, Ghana Health Service, Ghana

## Abstract

In this study, the seroprevalence of the intestinal worms *Taenia solium* and *Trichinella spiralis* in humans and pigs was assessed. A cross-sectional serological study design was performed. Blood samples were collected from 322 humans and 245 pigs used in the study. These were tested for markers of antibodies for *Taenia solium* and *Trichinella* spp. Demographic data such as sex, age, education, pig farming practices, and water source used were also obtained. An overall seroprevalence of 3.1% was recorded for *Taenia solium* in humans. There was also a statistical association between pig management system employed by pig farmers and seropositivity to *Taenia solium* (*p* = 0.005). Factors such as mode of waste disposal (*p* = 0.003) and water source used statistically correlated with *Taenia solium* seroprevalence among humans. For the pig samples, a *Taenia solium* seroprevalence of 24.9% was recorded. All the pig samples which tested positive for *Taenia solium* were reared on the free-ranged system. This study also recorded a seroprevalence of 0.31% for *Trichinella* spp. for humans and a seroprevalence of 4.5% for *Trichinella* spp. for pigs. Again, all the samples that showed serological evidence of *Trichinella* spp. among pigs came from those pigs which were raised on the free-ranged system. Proper pig management practice is a very important tool for controlling these intestinal parasites in both humans and animals. This study recommends public health education among the general public and good pig farming practices.

## 1. Introduction

Zoonotic enteric parasites are universal, presenting a significant public health challenge to human beings primarily due to an intimate relationship with domestic animals and wildlife and inadequate water, sanitation, and hygiene [1]. Pig-related zoonoses such as *Taenia solium* and *Trichinella spiralis* are implicated in areas with poor hygiene, water, and sanitation issues [2, 3]. These pig-related zoonotic diseases mostly affect people of the lower socioeconomic class, mostly in developing countries [4]. *Taenia solium* infections have been reported in several West African countries, although official statistics are often lacking [5]. Pork consumption has been recorded as a risk factor for *Taenia solium* and *Trichinella* infections [2]. Data on the prevalence of porcine *Taenia solium* are extremely scarce, unreliable, or underestimated in West Africa [5, 6]. Melki et al. [6] further argued that the available statistics are typically based on data obtained from “official” abattoirs and slaughterhouses, but in most countries in the developing world, majority of pigs are not slaughtered within approved slaughterhouses but often clandestinely without veterinary supervision. Coupled with that, the massive use of the extensive form of pig farming increases the exposure of pigs to contracting viable *Taenia solium* eggs [4, 7]. Lack of or inadequate meat inspections especially in unregistered pig slaughterhouses usually result in contaminated pork or pork products on the open markets [8]. However, Zoli et al. [5] argued that data about porcine cysticercosis obtained from pig slaughterhouses should be interpreted with caution. They argued that the data obtained from these meat inspections in these slaughterhouses are not representative of the real situation, since a large proportion of pigs, and certainly the cysticercotic pigs, are slaughtered outside the pig slaughterhouses [5]. In Ghana, some pigs are slaughtered either at home or on the farm and not at the pig slaughterhouse. There is also a general acceptance that pigs sent for slaughter are screened by tongue inspection by veterinarians, resulting in higher apparent prevalence on pig farms as compared to slaughterhouses [9]. According to Braae et al. [9], this approach causes bias issues in the studies done at slaughterhouses, which will underestimate the true prevalence of porcine cysticercosis, as pigs which carry high-intensity infections might have been eliminated from the sample. According to Onah and Chiejina [10], meat inspection usually underestimates the true prevalence of porcine cysticercosis where they found 20.5% of pigs with cysticercosis after detailed inspection of the carcass where the official figure of meat inspection reported only 3% of porcine cysticercosis. The detection of porcine cysticercosis usually based on the inspection of meat and tongue palpation is characterized by very low sensitivity which can lead to the underestimation of the true prevalence of porcine cysticercosis [11, 12]. For instance, in Burkina Faso, the first records of porcine cysticercosis around the year 2000 were based on meat inspection and a very low prevalence of 0.57% was recorded; however, later in 2011, a seroprevalence of 39.6% was recorded based on Ag-ELISA [13]. Bimi et al. [2] postulated that pork handlers and their households, together with people who prepare pork, stand a higher risk of tapeworm infection. From their study, men who were the pork sellers and their immediate family members stood a greater risk of infection. Occupational exposure is the reason why slaughterhouse workers and pork sellers who handle the offals and come into contact with the pig/pork stand a higher chance of contracting pig-related zoonoses like *Taenia solium* and *Trichinella* spp. when contaminated or raw meat is consumed. Studies on helminth infections among humans in Ghana are inadequate, especially among such groups as pig farmers, slaughterhouse workers, and pork sellers who are considered important players in the handling of pig/pork. Very few studies also exist on *Taenia solium* among pigs in Ghana, but these studies are concentrated on postmortem identification of viable cysts which might underestimate the true prevalence. Also, data on how the pigs were raised whether free-ranged or confined are not stated [2, 14, 15]. Despite its presence in several African countries and with the increase in pork consumption in Ghana, data on trichinellosis in humans is limited in Ghana. There has been no study in Ghana which has positively recorded *Trichinella* infections among humans. Also, not much studies have been done on the prevalence of *Trichinella* in pigs in Ghana, although Permin et al. [16] found no *Trichinella* larvae among cross-bred pigs in the Upper East Region of Ghana. This study was therefore conducted to determine the seroprevalence of these intestinal worms (*Taenia solium* and *Trichinella spiralis*) in humans in occupational exposure to pigs and also in pigs raised in the both the free-ranged and confined production systems.

## 2. Methods

### 2.1. Study Area

**Figure 1:**
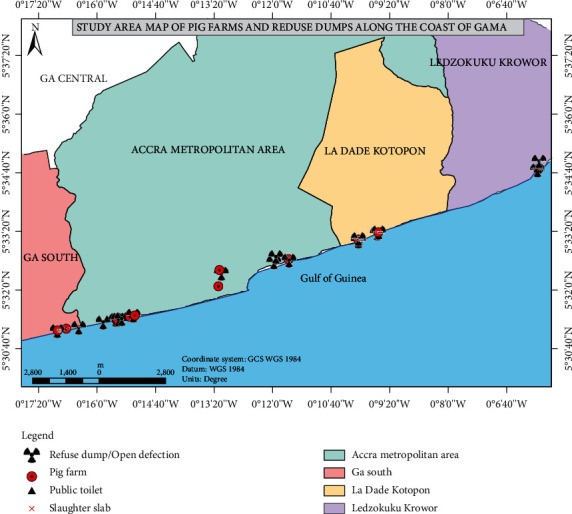
Map of Accra showing selected districts.

**Figure 2:**
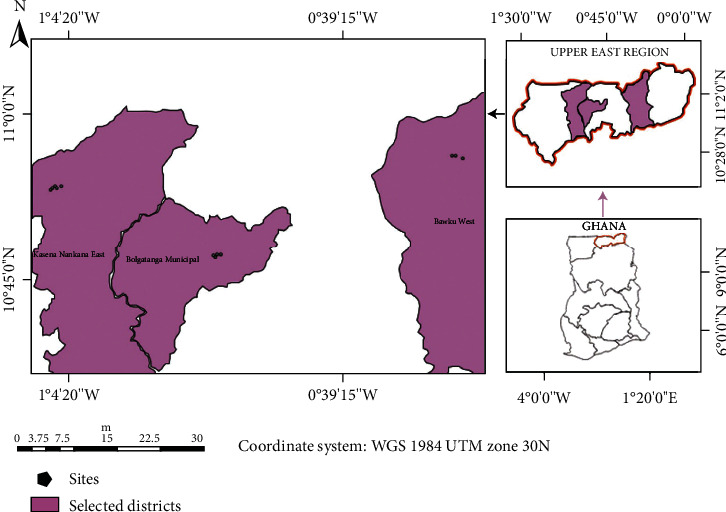
Map of the Upper East Region showing the selected study districts.

The study area purposively selected for this study has been noted as places of rigorous pig rearing and trading activities. Places selected were Ga South Municipality, Accra Metropolitan Assembly (AMA), Ladadekotopon Municipality, and Ledzokuku Municipality. These places are located along the Coast of the Greater Accra Metropolitan Area (GAMA) (Figure 1). These places have pigs housed in makeshift pens and mostly practice the free-ranged system of pig production. The Upper East Region located in the Northern part of Ghana was also purposively included in this study because it serves as a major conduit of pigs supplied to Southern Ghana (Figure 2).

### 2.2. Study Design

The study adopted a cross-sectional, serological study design in collecting data. The study period was between January and March 2018 among study participants from Accra who were pig farmers, slaughterhouse workers, pork sellers, and also among people living in adjoining households to pig farms. Serological studies were also done on pigs found within the selected communities during the same period. A similar cross-sectional serological survey was conducted among humans and pigs in the Upper East Region between July and August 2018.

### 2.3. Study Population

Groups which were involved in the pig/pork trade in Ghana were purposively selected for this study. The selected groups were identified as key players in the pig or pork trade in Ghana. The groups were pig farmers, slaughterhouse workers, and pork sellers. These were done to determine occupational exposure to pigs to determine the seroprevalence to the intestinal worms. To determine nonoccupational exposure to *Taenia solium* and *Trichinella spiralis* infections, households in close proximity to pig farms were recruited to be part of the study.

### 2.4. Sample Size and Selection Procedure

The calculation of the sample size was based on the formula put forward by Charan and Biswas [17]. The proportion assumed was 13.15% prevalence rate of pig-related zoonoses among those handling pig/pork as purported by Bimi et al. [2].

Thus, we have

(1) $$\begin{array}{c} n=\frac{Z^{2}p\left(1-p\right)}{d^{2}}, \end{array}$$

where *n* is the required sample size; *Z* is 95%confidence level = 1.96; *p* is the assumed proportion from similar studies which is 13.15% expressed in decimal = 0.1315; *q* = 1 − *p* is the probability of the event not occurring, in this case 1-0.1315; *d* is the 5% margin of error expressed in decimal = 0.05.

(2) $$\begin{array}{c} n=\frac{1.96^{2}\times 0.1315\left(1-0.1315\right)}{0.05^{2}}=n=\frac{0.43874}{0.0025}=175.5,\\ n≅176. \end{array}$$

Adding a 5% nonresponse rate, we have

(3) $$\begin{array}{c} \frac{5\times 176=9}{100},\\ n=176+9=185, \end{array}$$

for human samples for this study.

For the pig samples used for this study, the proportion assumed was 18.8% prevalence rate as purported by Bimi et al. [2].

Thus, we have

(4) $$\begin{array}{c} \frac{n=Z^{2}p\left(1-p\right)}{d^{2}}, \end{array}$$

where *n* is the required sample size; *Z* is 95%confidence level = 1.96; *p* is the assumed proportion from similar studies which is 18.8% expressed in decimal = 0.188; *q* = 1 − *p* is the probability of the event not occurring, in this case 1-0.188 = 0.812; *d* is the 5% margin of error expressed in decimal = 0.05.

(5) $$\begin{array}{c} n=\frac{1.96^{2}\times 0.188\;\left(1-0.188\right)}{0.05^{2}}=n=\frac{0.5864}{0.0025}=234.58,\\ n≅235. \end{array}$$

Adding a 5% nonresponse rate, we have

(6) $$\begin{array}{c} \frac{5\times 176}{100}=9,\\ N=235+9=244, \end{array}$$

where ≅245 pig samples were sampled for this study.

### 2.5. Data Collection Methods

#### 2.5.1. Blood Sample Collection and Processing

Five (5) ml of blood samples was collected from both humans and pigs into the gel separation vacutainer tubes. The blood samples were appropriately labelled and transported on ice to the National Public Health Reference Laboratory, Korle-Bu, Ghana. There, the blood samples were allowed to clot and were centrifuged for 10 minutes. The serum was then placed in cryovials for processing.

#### 2.5.2. Enzyme-Linked Immunosorbent Assay (ELISA)

Samples collected for this study were serologically examined for the evidence of markers of *Teania solium* and *Trichinella spiralis* in both humans and pigs. The purpose of such a test was to detect serum antibodies or antibody-like substances that appear specifically in association with certain diseases.

The Novatec Immundiagnostica GMBH *Taenia solium* IgG (Lot number: TAE-061; sensitivity 94%; specificity 95%; Novatec Immundiagnostica, Germany) was the ELISA kit used for the serological testing of *Taenia solium* in humans, and porcine sera were analyzed for the detection of antibodies against *Taenia solium* (IgG) using the Novatec Vetline *Taenia solium* (Lot number: TAEVT-061-1; sensitivity 95%; specificity 95%; Novatec Immundiagnostica, Germany). The Novatec Immundiagnostica GMBH *Taenia solium* IgG is intended for the qualitative determination of IgG class antibodies against *Taenia solium* in human serum or plasma. The VetLine *Taenia* ELISA is also intended for the qualitative determination of IgG antibodies against *Taenia* in veterinary mammalian serum. The VetLine contains 12 break-apart 8-well snap-off strips coated with *Taenia* antigens. This ELISA kit makes use of the larval extract of pig tapeworm as an antigen, and this extract was fractionated.

The manufacturer's instructions were followed in testing serum samples for the presence of exposure to *Taenia solium*. Before assaying, all samples were diluted 1 : 100 with the IgG Sample Diluent.

The IBL International GMBH *Trichinella spiralis* IgG ELISA was the ELISA kit used for the serological testing of *Trichinella spiralis* in humans. The IBL International GMBH *Trichinella spiralis* IgG ELISA (Lot number: TRIG-065; sensitivity 100%; specificity 95%; IBL International, Germany) is intended for the qualitative determination of IgG class antibodies against *Trichinella spiralis* in human serum or plasma (citrate, heparin). The *Trichinella spiralis* ELISA contains 12 break-apart 8-well snap-off strips coated with *Trichinella spiralis*. The manufacturer's instructions were followed in testing serum samples for the presence of exposure to *Trichinella spiralis*. Before assaying, all samples were diluted 1 : 100 with the IgG Sample Diluent.

Serum samples from pigs also were analyzed for the detection of antibodies against *Trichinella spiralis* (IgG) using the PrioCHECK Porcine *Trichinella* Ab 450 (Lot number: T170301L; sensitivity 97%; specificity 98%; Prionics, Switzerland). The PrioCHECK *Trichinella* Ab is used for in vitro detection of IgG antibodies against *Trichinella* spp. in serum and meat juice of pigs. The manufacturer's instructions were strictly followed in testing pig serum samples for the presence of exposure to *Trichinella* spp. The following steps were applied:

- *Sample dilution*: the control sample was reconstituted by adding 150 *μ*l of demineralized water. This was mixed by vortexing thoroughly and inverting the vial several times. A dummy plate was used for the first sample dilution. The positive controls were designated for wells A1 and B1 of the dummy plate to which 10 *μ*l of these positive controls was added. Weak positive controls were assigned to wells C1 and D1 of the dummy plate and to which 10 *μ*l of the weak positive controls was added. Ten (10) *μ*l of negative controls was added to wells E1 and F1 of the dummy plate. Ten (10) *μ*l of serum samples was then added to the remaining wells of the dummy plate. Ninety (90) *μ*l of sample diluent was added to each well of the dummy plate, and it was mixed by pipetting up and down for 5 times. To the test plate, 80 *μ*l of the sample diluent was added to each well. Twenty (20) *μ*l of the diluted samples and 20 *μ*l of the controls were transferred from the dummy plate to the test plate and mixed by pipetting up and down 5 times
- *Sample incubation*: the samples were then incubated on the test plate for 30 ± 1 minutes at room temperature (22°C ± 3). The test plate was washed four times with 300 *μ*l 1x wash fluid working solution using the Biotek Microplate plate washer
- *Conjugate incubation*: the conjugate was first diluted before use by the proportion of 400 *μ*l of conjugate to 11.6 ml conjugate diluent for one full plate of 96 wells. One hundred (100) *μ*l of the diluted conjugate was added to each well of the test plate. The test plate was afterwards incubated at 22°C ± 3 for 30 ± 1 minutes. The test plate was then washed four times with 300 *μ*l 1x wash fluid working solution using the Biotek Microplate plate washer
- *Detection*: one hundred (100) *μ*l of the chromogen (TMB) substrate was added to each well on the test plate. The test plate was incubated for 15 ± 1 minutes at 22°C ± 3. One hundred (100) *μ*l of the stop solution was added to each well of the test plate. The addition of the stop solution started 15 ± 1 minutes after the first well was filled with chromogen (TMB) substrate solution. The stop solution was added in the same order as the chromogen (TMB) substrate solution was dispensed. The colour of the positive controls changed from blue to yellow. The test plate was shaken shortly (5-10 s) on an orbital shaker. The result of the test plate was read at 450 nm within 15 minutes by using a Biotek plate reader

For *Taenia solium*, results were interpreted as positive if it was greater than 11 NTU (U). Positive results mean that antibodies against the pathogen were present. Results were interpreted as negative if it was less than 9 NTU (U). Thus, the sample contained no antibodies against the pathogen. For *Trichinella spiralis*, all results that were above or equal to the cut-off of 15 PP were considered positive. Results recorded which were below the cut-off of 15 percentage positivity (PP) were considered negative.

### 2.6. Data Analysis

Questionnaire and laboratory data were entered into a spreadsheet (Excel; Microsoft, Redmond, WA, USA) and analyzed using STATA software version 14.2 (StataCorp LP, USA). Descriptive measures such as the mean, frequencies, and percentages were employed to describe the variables under study. Prevalence was calculated as all individuals who showed serological evidence of infection over the total number tested as a percentage.

### 2.7. Ethical Considerations

The study protocol was approved by the Ethics Committee for Basic and Applied Sciences (ECBAS). This study collected data (blood samples) from both humans and pigs between December 2017 and August 2018. The study protocol (ECBAS 010/17-18) was carefully and verbally explained to each participant in a language they understood and each participant was assured of confidentiality. Each participant also had the chance to ask questions at each point of the study to which answers were provided. After voluntary agreement to participate, participants consented and were at liberty to withdraw from the study at any point of the study. Literate participants were given written forms to read and sign. Illiterate participants were asked to provide a literate witness who signed on their behalf before they were asked, in the presence of their witness to thumbprint the consent form.

## 3. Results

### 3.1. Seroprevalence of *Taenia solium* among Humans in Accra and Upper East

From the study, the overall seroprevalence for *Taenia solium* recorded for humans in both Accra and the Upper East Region was 3.1% (10/322). However, there was no statistical relationship between the location of where the humans came from and seropositivity to *Taenia solium* and cysticercosis (*p* = 0.656) (Table 1).

#### 3.1.1. Age Group

Seropositivity to *Taenia solium* was not correlated with the age group in this study (*p* = 0.584). The highest seroprevalence was recorded among age groups 41-50 years and 51-60 years (Table 2).

#### 3.1.2. Occupation

Occupationally related exposure to pigs such as slaughterhouse workers, pig farmers, and pork sellers had no statistical relationship with seropositivity to *Taenia solium* (*p* = 0.155) (Table 2).

#### 3.1.3. Mode of Waste Disposal

The highest seroprevalence was recorded among respondents who practiced crude or open dumping (14.6%). There was a statistical relationship between waste disposal and seropositivity to *Taenia solium* (*p* = 0.003) (Table 2).

#### 3.1.4. Water Source Used

The water source used by respondents was statistically associated with *Taenia* seroprevalence (*p* = 0.025). All the respondents who tested positive for *Taenia solium* relied on community standpipe for their water use needs (6.2%). Respondents who used other water sources all tested negative for *Taenia solium* (Table 2).

#### 3.1.5. Pig Management System

Only pig farmers who raised pigs on the free-ranged system tested positive to *Taenia solium* in the Upper East. The highest *Taenia solium* seroprevalence was recorded among pig farmers from Accra who practiced the free-ranged system (Table 2).

**Figure 3:**
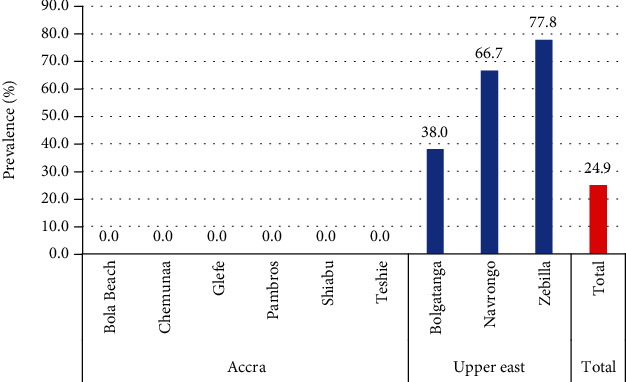
Prevalence of *Taenia* among pigs in Accra and Upper East by location.

All the pig samples which showed serological evidence of infection to *Taenia solium* came from pigs selected from the Upper East Region (Figure 3).

From this study, the overall seroprevalence of *Taenia solium* among pigs in both Accra and the Upper East Region was 24.9% (61/245) (Table 3). There was a statistical association between location and seropositivity to *Taenia solium* among pigs (*p* = 0.005) (Table 3). There was a strong statistical association between municipality and *Taenia solium* seropositivity in pigs (*p* < 0.001) (Table 3).

There was also a statistical association between the pig management system employed by pig farmers and seropositivity to *Taenia solium* in pigs (*p* = 0.005) (Table 3).

### 3.2. Seroprevalence of *Trichinella* spp. among Humans

An overall seroprevalence of 0.31% was recorded among human respondents from this study, and there was no statistically significant difference between Accra and Upper East (Table 4).

From this study, the overall seroprevalence of *Trichinella* spp. among pigs in both Accra and the Upper East Region was 4.5% (11/245) (Table 5). There was no statistical association between location and seropositivity to *Trichinella* spp. among pigs (*p* = 0.336) (Table 5). All the pig samples that tested positive for *Trichinella* spp. came from Bolgatanga in the Upper East Region. All the other locations recorded 0% seroprevalence. Again, all the samples that showed serological evidence of *Trichinella* spp. among pigs came from those pigs who were raised on the free-ranged system (Table 5).

## 4. Discussion

### 4.1. Seroprevalence of *Taenia solium* in Humans and Pigs

This study recorded very low prevalences for *Taenia solium* and *Trichinella* spp. in both humans and pigs across the study sites. This might be due to the fact that here in Ghana we mostly overcook our meat products. A *Taenia solium* seroprevalence of 3.1% in humans was recorded for this present study. This is markedly lower than the *Taenia solium* prevalence of 13.15% recorded by Bimi et al. [2] among the stool samples of people of Northern Ghana. However, the seroprevalence of 3.1% for *Taenia solium* was higher than another study in the Kintampo North Municipality where a prevalence of 1.1% was recorded among study participants [18]. The finding of this present study compares favourably with 3.1% seroprevalence among respondents in West Cameroon [19]. In a systematic review of the seroprevalences of *Taenia solium* human cysticercosis, prevalences of 7.30%, 4.08%, and 3.98% were recorded for circulating *Taenia solium* antigens for Africa, Latin America, and Asia, respectively [20]. Similarly, seroprevalences of 17.37%, 13.03%, and 15.68% for *Taenia solium* antibodies were reported for Africa, Latin America, and Asia, respectively [20]. This present study recorded a seroprevalence of 24.9% for *Taenia solium* among pigs from the study sites. There was also an association between where the pigs came from and seropositivity to *Taenia solium*/cysticercosis. All the pigs which showed serological markers for *Taenia solium* came from the Upper East Region. This finding is not surprising because the Upper East Region is noted to have the worst sanitation coverage in Ghana with many open defecation sites. This means that these pigs have access to more open defecation sites. The seroprevalence of 24.9% recorded in this study is corroborated by a study in Uganda which found a slightly higher prevalence of 25.7% among pigs [21]. Permin et al. [16], however, found a lower *Taenia solium* prevalence of 11.7% when they researched pigs from the same region, i.e., the Upper East Region. Similarly, Bimi et al. [2] recorded *Taenia* cysts in 18.8% among pigs slaughtered for consumption in the Northern Region of Ghana. Through their stochastic model, Assana et al. [22] predicted the expected prevalence of *Taenia solium* porcine cysticercosis in Ghana at 25.6%. In another study in pig slaughterhouses in Kumasi, Atawalna and Mensah [23] also found a porcine cysticercosis prevalence of 2.31%, lower than the 24.9% recorded in this present study. Atawalna and Mensah [23] argue that porcine cysticercosis mostly is not related to clinical signs of disease and that most of the cases are detected during routine postmortem inspections during slaughter. Another study in the Adamawa state in Nigeria also recorded a similarly lower porcine cysticercosis prevalence of 3.2% [24]. However, a higher prevalence rate of 46% has been recorded among pigs in the Jos Metropolis in Nigeria [25]. Uganda has up to 45% of the pig population infected with cysticercosis in some villages, and cysticercosis prevalences in pigs in many countries stand at 10% [26]. Assana et al. [22] estimated that 73.31% of pigs in Ghana are at risk of *Taenia solium* porcine cysticercosis.

### 4.2. Seroprevalence of *Trichinella* spp. among Humans and Pigs

This is the first study in Ghana to report of positive serological evidence of *Trichinella* spp. infections in humans. Seroprevalences of 0.31% and 4.5% for *Trichinella* spp. in both humans and pigs, respectively, were recorded. Trichinellosis incidence rates of 0.02% and 0.04% have been found in studies in Ethiopia [27]. Studies around the world have recorded similarly low prevalences of *Trichinella* in humans. For example, De-La-Rosa et al. [28] found seroprevalences between 1.0% and 1.9% when they studied prevalence and risk factors associated with serum antibodies against *Trichinella spiralis* in Mexico. In rural communities in Chile, Contreras et al. [29] found a seroprevalence of 1.5% among their study participants. Also, the seroprevalence of 0.31% recorded in this present study was lower than the 19.1% *Trichinella* seroprevalence recorded among humans in Northern Laos [30]. Okello et al. [31], however, found a higher seroprevalence of 58.6% for *Trichinella* spp. in human samples from Laos People's Republic. One major drawback in knowing the true prevalence of *Trichinella* infections is primarily due to the lack of accurate data on cases. De-La-Rosa et al. [28] postulated that trichinellosis in humans is likely present in subtle endemic form due to the fact that it can remain asymptomatic and also produces similar symptoms like typhoid fever which can lead to misdiagnosis especially in resource-poor settings.

The 4.5% seroprevalence for *Trichinella* spp. recorded in this study came from pigs sampled from the Upper East Region of Ghana. Permin et al. [16] found 0% prevalence when they studied cross-bred pigs from the same region. Gamble and Bush [32] also found a very low prevalence of 0.013% of *Trichinella* spp. in pigs from the United States. Data on *Trichinella* spp. in pigs is lacking in Ghana. Only two studies from sub-Saharan African countries that found *Trichinella*-specific antibodies among domestic pigs have been published, reporting seroprevalences of 40% [33] and 11% [34]. Conlan et al. [30] also isolated *Trichinella* larvae from 2.1% of sampled pigs from Northern Laos, which is lower than the seroprevalence of 4.5% recorded in this present study. Another study found a higher *Trichinella* spp. seroprevalence of 23.4% among pigs in Laos People's Republic [31]. Similarly, a higher seroprevalence than what was recorded in this present study (6.9%) was found among domestic pigs in Central and Eastern Uganda [35] and also in pigs (9.0%) in endemic regions in Argentina [36]. Pozio [37] argues that the low prevalence of *Trichinella* spp. recorded among pigs in several studies despite millions of pigs tested yearly and people getting infected with *Trichinella* spp. means that the wrong pigs are usually tested. The emphasis should be placed on free-ranged scavenging pigs and not those reared under a controlled environment.

While the major risk factor for human trichinellosis is the consumption of contaminated meat or poorly cooked meat, the risk factors related to the presence of *Trichinella* spp. in pigs are the ingestion of infectious larvae by the scavenging behaviour of animals especially pigs. From the current study, all the pigs that showed serological markers of *Trichinella* spp. were those pigs reared on a free-ranged basis. This means that these pigs have access to open defecation sites, refuse dumps, and other contaminated sites as these pigs came from the Upper East Region in Ghana with the worst sanitation record. The low prevalences of *Trichinella* spp. in both humans and pigs recorded in this present study could be attributed to the lack of official data on pig slaughterhouses which might inform the prevalence. The situation has quite worsened due to home slaughter without veterinary supervision. Proper and adequate meat inspection is required to control the presence of *Trichinella* spp. in both humans and pigs. The pigs that tested positive for markers of *Trichinella* spp. all came from the Upper East Region. These communities in the Upper East Region are major supply conduits of pigs to Accra. This makes the role played by supervisory bodies like Veterinary Officers, Municipal/District Assemblies, and other allied regulatory bodies even more necessary. These pigs pass through several checks before they finally arrive in Accra.

## 5. Conclusion

Till date, no outbreak of *Taenia solium* and *Trichinella* spp. have been recorded in Ghana. Despite this, all the risk factors for their occurrence are present: increase in pork consumption, presence of free-ranging pigs, poor sanitation conditions in the country, and presence of open defecation sites. A One Health approach comprised of veterinary officers, environmental health officers, medical experts, and all aspects of social scientists is needed for zoonotic parasites of pigs like *Taenia solium* taeniasis and *Trichinella* spp. to be controlled.

## Figures and Tables

**Table 1:** Seroprevalence of *Taenia solium* in humans and test of association between location and *Taenia solium* seropositivity.

Location	*N* (%)	Positive (%)	Negative (%)	Prevalence (%)	ToP	*z*	Std. Err.
Accra	238 (73.9)	8 (3.4)	230 (96.6)	3.4	0.0336	0.45	0.011
Upper East	84 (26.1)	2 (2.4)	82 (97.6)	2.4	0.0238		0.017
Total	322 (100.0)	10 (3.1)	312 (96.9)	**3.1**			

Fisher′s exact = 0.1983. *p* value = 0.656. ^∗^ToP: two-sample test of proportion.

**Table 2:** Association between *Taenia solium* seropositivity and predictor variables.

Variables	Negative (%)	Positive (%)	Chi-square (*p* value)
Sex			
Male	209 (95.9)	9 (4.1)	2.3467 (0.126)
Female	103 (99.0)	1 (1.0)	
Age group (years)			
Under 20	17 (94.4)	1 (5.6)	3.7622 (0.584)
20-30	54 (98.2)	1 (1.8)	
31-40	99 (98)	2 (2.0)	
41-50	82 (96.5)	3 (3.5)	
51-60	41 (93.2)	3 (6.8)	
61 and above	19 (100.0)	0 (0.0)	
Occupation			
Slaughterhouse worker	26 (100.0)	0 (0.0)	5.2332 (0.155)
Farmer	131 (97.0)	4 (3.0)	
Community members	97 (94.2)	6 (5.8)	
Pork seller	58 (100.0)	0 (0.0)	
Mode of waste disposal			
Collection trucks	24 (100.0)	0 (0.0)	26.8043 ^∗^(**0.003**)**+**
Community collection point	44 (100.0)	0 (0.0)	
Community refuse dump	51 (96.2)	2 (3.8)	
Dumped into the sea	47 (97.9)	1 (2.1)	
Household collection to community dumpsite	9 (100.0)	0 (0.0)	
Open dumping	41 (85.4)	7 (14.6)	
Wastes dried and burnt	39 (100.0)	0 (0.0)	
Wastes gathered and dried	57 (100.0)	0 (0.0)	
Water source used			
Borehole	26 (100.0)	0 (0.0)	10.1931 ∗**(0.025)**^**+**^
Community borehole	66 (100.0)	0 (0.0)	
Community standpipe	152 (93.8)	10 (6.2)	
In-house pipe borne	68 (100.0)	0 (0.0)	
Municipality			
AMA	160 (97.0)	5 (3.0)	11.6076 (0.071)
Bawku West	31 (100.0)	0 (0.0)	
Bolgatanga	28 (93.3)	2 (6.7)	
Kasena-Nankana East	23 (100.0)	0 (0.0)	
LADMA	21 (87.5)	3 (12.5)	
LEKMA	11 (100.0)	0 (0.0)	
Weija-Gbawe	38 (100.0)	0 (0.0)	
Pig management system			
Free-ranged	92 (96.8)	3 (3.2)	0.0424 (0.837)
Confined	39 (97.5)	1 (2.5)	

^∗^Significant at *p* < 0.05; ^+^Fisher's exact *p* values (some cell frequencies < 5).

**Table 3:** Association between predictor variables and *Taenia* prevalence among pigs.

*Taenia solium* (IgG)	Negative			Positive			Total			
	*N*	Col (%)	Row (%)	*N*	Col (%)	Row (%)	*N*	Col (%)	Row (%)	Prevalence (%)
*Municipality*										
AMA	58	31.5	100	0	0.0	0.0	58	23.7	100	0.0
Bawku West	2	1.1	22.2	7	11.5	77.8	9	3.7	100	77.8
Bolgatanga	85	46.2	62	52	85.2	38	137	55.9	100	38.0
Navrongo	1	0.5	33.3	2	3.3	66.7	3	1.2	100	66.7
LEKMA	22	12	100.0	0	0.0	0.0	22	9.0	100	0.0
Weija-Gbawe	16	8.7	100.0	0	0.0	0.0	16	6.5	100	0.0
Total	184	100.0	75.1	61	100.0	24.9	245	100	100	**24.9**
Pearson′s Chi^2^(5) = 60.5772*p* ≤ 0.001										
*Pig management system*										
Confined	22	12	100	0	0	0	22	9.0	100	0.0
Free-ranged	162	88	72.6	61	100	27.4	223	91.0	100	27.4
Total	184	100	75.1	61	100	24.9	245	100.0	100	**24.9**
Pearson′s Chi^2^(1) = 8.0130*p* = 0.005										

**Table 4:** Prevalence of *Trichinella* spp. among humans.

Location	*N* (%)	Positive (%)	Negative (%)	Prevalence (%)
Accra	238 (73.9)	1 (0.4)	237 (99.6)	0.4
Upper east	84 (26.1)	0 (0.0)	84 (100.0)	0.0
Total	322 (100.0)	1 (0.3)	321 (99.7)	**0.31**

Fisher′s exact = 0.3540; *p* value = 0.552.

**Table 5:** Seroprevalence of *Trichinella spiralis* and predictor variables among pigs.

*Trichinella* spp.	Negative			Positive			Total			
	*N*	Col (%)	Row (%)	*N*	Col (%)	Row (%)	*N*	Col (%)	Row (%)	Prevalence
*Municipality*										
AMA	58	24.8	100	0	0	0	58	23.7	100	0.0
Bawku West	9	3.8	100	0	0	0	9	3.7	100	0.0
Bolgatanga	126	53.8	92	11	100	8	137	55.9	100	8.0
Navrongo	3	1.3	100	0	0	0	3	1.2	100	0.0
LEKMA	22	9.4	100	0	0	0	22	9	100	0.0
Weija-Gbawe	16	6.8	100	0	0	0	16	6.5	100	0.0
Total	234	100	95.5	11	100	4.5	245	100	100	**4.5**
Pearson′s Chi^2^ (5) = 9.0792; *p* = 0.106										
*Pig management system*										
Confined	22	9.4	100	0	0	0	22	9	100	0.0
Free-ranged	212	90.6	95.1	11	100	4.9	223	91	100	4.9
Total	234	100	95.5	11	100	4.5	245	100	100	**4.5**
Pearson′s Chi^2^ (1) = 1.1362; *p* = 0.286										

## Data Availability

The data used in this work is available upon request from the corresponding author.
